# Recurrent corneal melting in the paraneoplastic pemphigus associated with Castleman’s disease

**DOI:** 10.1186/s12886-016-0280-7

**Published:** 2016-07-12

**Authors:** Haijun Gong, Shiyou Zhou, Yuxin Hu, Yuqin Lan, Hong Zeng, Liangchun Wang, Qingyu Liu, Mei Wang

**Affiliations:** Department of Ophthalmology, Sun Yat-sen Memorial Hospital of Sun Yat-sen University, Guangzhou, 510120 China; Zhongshan Ophthalmic Center, Sun Yat-sen University, State Key Laboratory of Ophthalmology, Guangzhou, 510120 China; Department of Pathology, Sun Yat-sen Memorial Hospital of Sun Yat-sen University, Guangzhou, 510120 China; Department of Dermatology, Sun Yat-sen Memorial Hospital of Sun Yat-sen University, Guangzhou, 510120 China; Department of Radiology, Sun Yat-sen Memorial Hospital of Sun Yat-sen University, Guangzhou, 510120 China

**Keywords:** Corneal melting, Paraneoplastic pemphigus, Castleman’s disease

## Abstract

**Background:**

The ocular presentation of Castleman’s disease (CD)-associated paraneoplastic pemphigus (PNP) has rarely been reported. In this report, we describe a young patient with CD-associated PNP who had recurrent corneal ulceration in addition to cicatrizing conjunctivitis.

**Case presentation:**

We describe a case of 23-year-old male with mucocutaneous erosion and conjunctival injection and erosion who was found to have PNP. Pelvic hyaline-vascular CD was detected and completely excised. The mucocutaneous lesions improved postoperatively. Two years after pelvic surgery, the patient gradually developed conjunctival symblepharon in both eyes and pterygium in the right eye. The patient then underwent a successful exclusion of the symblepharon, an excision of the pterygium and an amniotic membrane transplantation in the right eye. However, after 6 months, he experienced an aseptic corneal ulcer and recurrent pterygiumin the right eye. After treatment with systemic and local immunosuppressive medications, the corneal ulcer gradually healed and remained stable.

**Conclusion:**

Corneal ulceration and melting, in addition to conjunctivitis, as a complication of CD-associated PNP, can be successfully managed with systemic and local immunosuppressants.

## Background

Paraneoplastic pemphigus (PNP) is a new autoimmune mucocutaneous blistering disease associated with neoplasms, such as Castleman’s disease (CD) [1–3]. PNP is characterized by distinctive clinical symptoms and signs, such as severe and painful mucosal erosions and polymorphous skin lesions [1, 2].

Cicatrizing conjunctivitis is the main ocular presentation of PNP [4], and only one case of bilateral corneal melting has been reported in a patient with PNP and a peripheral neuronal shaft tumor [5]. However, the ocular involvement of CD-associated PNP has rarely been reported. In this report, we describe a young patient with CD-associated PNP who developed recurrent corneal ulceration in addition to conjunctivitis after the resection of the tumor.

## Case presentation

In November 2011, a 23-year-old man presented with persistent, painful oral erosions resistant to systemic corticosteroid and antibiotic treatment. Conjunctival congestion and erosion was also present, but there were no other ocular abnormalities on slit-lamp and direct fundoscopic examinations. Local antibiotic eye drops and ointment were given. Erosion on the fingers in both hands appeared later.

**Fig. 1:**
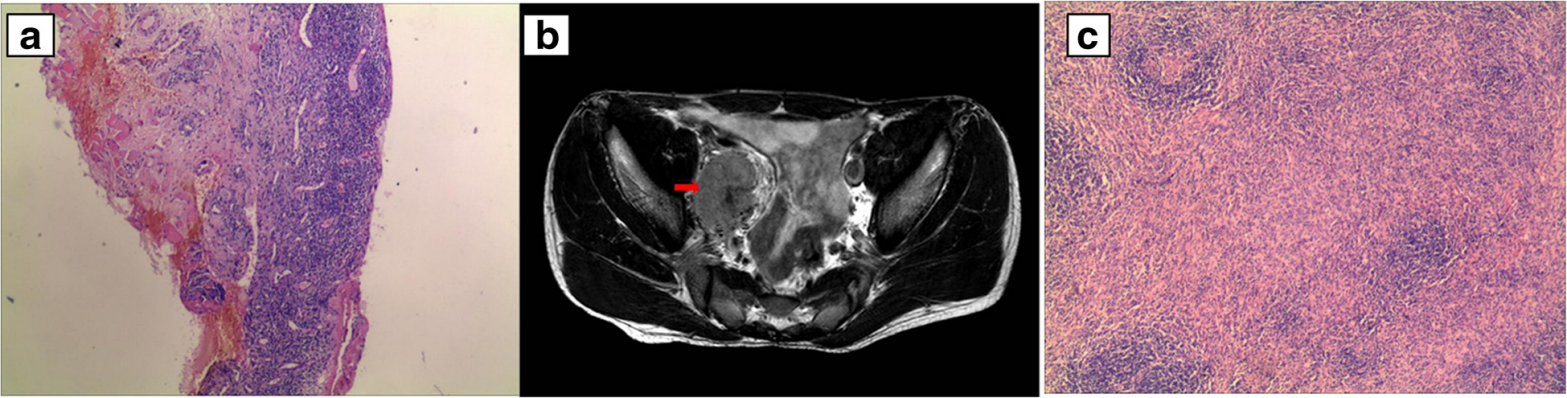
The imaging and pathological features. **a** Formation of small mucous membraneulcers, lichenoid interface infiltration and keratinocytenecrosis (hematoxylin-eosin stain; original magnification × 40). **b** Pelvic MRI (the *red arrow* indicates the 4.9 × 3.7 × 5.1 cm tumor). **c** Histology revealed small-sized follicles with hyalinized germinal centers and “onionskin” arrangement mantle zone lymphocytes in addition to interfollicular hypervascularity without plasma cell sheets. (hematoxylin-eosinstain; original magnification × 100)

The serum markers for rheumatic diseases, cancer, HIV and syphilis infection were negative. A histological examination of a buccal mucosal biopsy with hematoxylin-eosin staining showed a small mucous membrane ulcer formation, lichenoid interface infiltration and keratinocyte necrosis (Fig. 1a). Serum antibodies against desmoglein (Dsg)-3 were detected by ELISA, but anti-Dsg-1 antibodies were not found. Indirect immunofluorescence (IIF) demonstrated IgG autoantibodies directed toward rat bladder transitional cell epithelium. The patient was diagnosed with PNP according the criteria for the diagnosis of PNP proposed by Camisa and Helm [6].

**Fig. 2:**
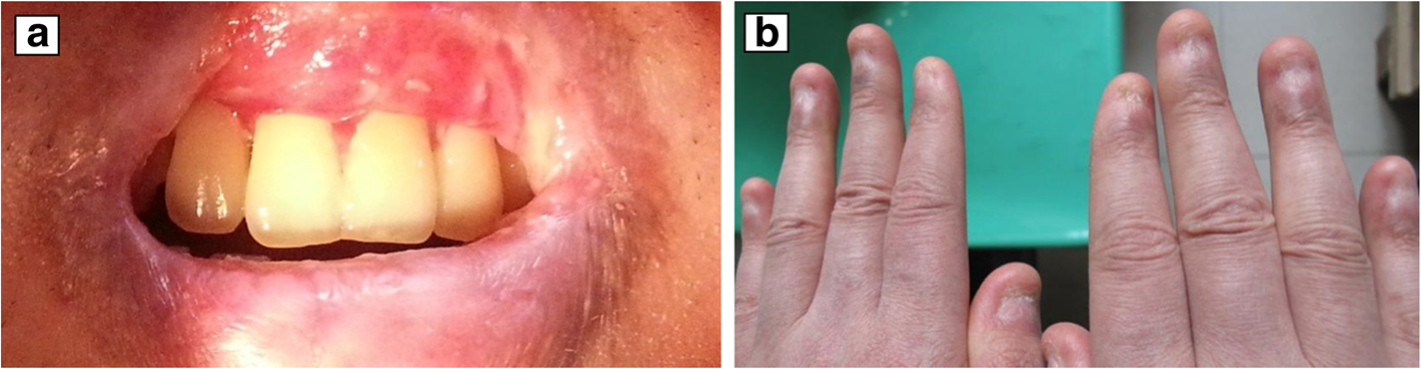
The mucosa and skin presentation. **a** The oral erosion resolved, leaving an oral mucosal scar. **b** The nail erosions were resolved, and the nail plates were lost bilaterally

An MRI revealed a pelvic mass (4.9 × 3.7 × 5.1 cm) that was located in the area of the right iliac vessels, and it had well-defined border (Fig. 1b). The patient subsequently underwent a complete excision of the pelvic mass after receiving intravenous gamma globulin (20 g daily for 3 days). A histopathologic examination revealed a lymph node specimen constituted by small-sized follicles with regressed germinal centers and “onion skin” arrangement mantle zone lymphocytes. The germinal centers were almost totally replaced by endothelial and dendritic cells. Blood vessels with hyalinized walls also penetrated into regressed germinal centers forming a hyaline-vascular lesion. The inter follicular stroma abounded in numerous high endothelial venules with sclerotic walls. No normal lymphoid tissue or lymph node sinus was recognized circumferentially. No plasma cell sheets were detected (Fig. 1c). Therefore, the hyaline vascular variant of CD was diagnosed. Then, after the conjunctival erosion was cured and the injection was alleviated, local antibiotic treatment ceased 4 weeks postoperatively. The patient’s mucosal and nail lesions had gradually improved by 5 months after the surgery (Fig. 2a, b). Postoperative management consisted of oral prednisone (50 mg daily) and cyclosporine (150 mg daily), which were gradually tapered. IgG antibody direct to rat bladder transitional cell epithelium was examined for several times, the titer decreased significantly from 1:320 before pelvic surgery to 1:80 2 years after surgery, and varied from 1:10 to 1:80 during the following follow-up. While anti-Dsg3 antibody was not routinely tested since it is not critical among the antibody spectrum of PNP.

**Fig. 3:**
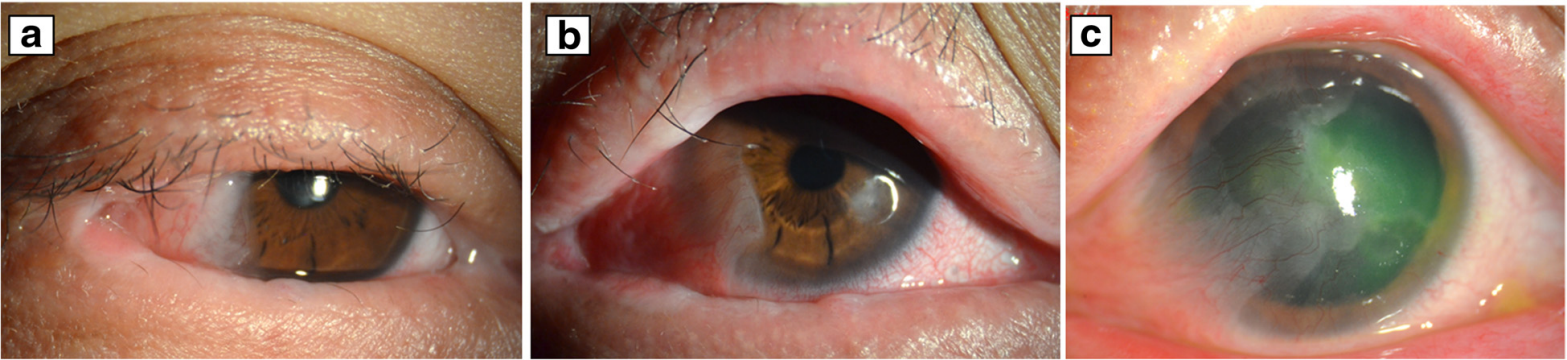
The ocular presentation. **a** The right conjunctiva gradually developed symblepharon, pterygium and hypophasis. **b** Six months after ocular surgery, recurrent pterygium and a 2 mm-diameter aseptic corneal ulcer developed in the nasal paracentral non-surgical area in the right eye. **c** To date, corneal melting has been stable; in the right eye, the epithelium is intact, and mild conjunctival congestion and temporal pterygium are present

In August 2013, 2 years after the pelvic surgery, the patient was admitted to our hospital’s Department of Ophthalmology with a symblepharon of the lateral canthus in the both eyes and temporal pterygium in the right eye (Fig. 3a). The size of the pterygium, measured from limbus to the head of pterygium, was 4 mm at its longest diameter. Both the anterior chamber and fundus examinations were normal. Considering the conjunctival inflammation inactive, the surgery, including an exclusion of the symblepharon, an excision of the pterygium, and amniotic membrane grafting, was successively performed in the right eye. Tobramycin-dexamethasone eye drops (TobraDex; Alcon Laboratories, Inc., Fort Worth, TX, USA) and nonsteroidal anti-inflammatory eye drops (Pranoprofen eye drops; Senju Pharmaceutical Co, Ltd, Osaka, Japan) were administered postoperatively, tapered gradually and stopped 4 weeks postoperatively.

In February 2014, 6 months after ocular surgery, the patient complained of pain and decreased vision in the right eye. A slit-lamp examination revealed conjunctival congestion, recurrent pterygium and a 2 mm-diameter aseptic corneal ulcer in the nasal-paracentral non-surgical area (Fig. 3b). There were no signs of ciliary injection and no conjunctival secretions. Initially, the administration of TobraDex eye drops every 2 h per day for 1 week and a bandage contact lens for 3 day failed to treat the corneal ulcer. Then, 0.1 % tacrolimus eye drops (prepared by the hospital) were prescribed instead of corticosteroid eye drops. The corneal ulcer gradually healed, with the remission of eye pain and an improvement in vision. However, once treatment with the tacrolimus eye drops was discontinued, the corneal ulcer recurred. Therefore, a topical 0.1 % tacrolimus twice per day and a 1 % cyclosporine ointment were prescribed. The same therapy was also applied to the left eye to treat a minor recurrence in the left eye. To date, the corneal melting has been stable for 6 months, with an intact epithelium, local corneal thinning and temporal pterygium in the right eye (Fig. 3c), as well as mild bilateral conjunctival congestion and posterior capsular lens opacity. A slight symblepharon developed in the left eye. The posterior capsular lens opacity was thought to be due to long-term systemic corticosteroid therapy, while the IOP in both eyes was normal. The patient was followed closely.

Currently, he is on systemic methylprednisone 16 mg daily, thalidomide 75 mg twice per day and methotrexate 10 mg weekly to treat oral erosions and ocular lesions. No evidence of tumor recurrence has been found on multiple follow-up pleural and abdominal CT and MRI scans. The results of routine laboratory testing have been normal. Autoantibodies against rat bladder transitional epithelium remain positive (with a low titer of 1:10).

## Discussion

Limited studies of CD-associated PNP have been reported. In addition, the ocular presentation has not been reported relating CD-associated PNP. Conjunctival injections and erosions have been mentioned in some cases of CD-associated PNP [7–9]. As far as we know, this report is the first that examines unilateral corneal ulceration and melting, in addition to cicatrizing conjunctival lesions, in CD-associated PNP.

The most common causes of corneal melting are infection, vitamin A deficiency, collagen vascular disease, severe Sjögren’s syndrome and neurotrophic keratitis [10]. Each of these causes could be excluded in this patient, based on his medical history, his clinical symptoms and signs, and the results of the laboratory studies. Thus, an immunologic mechanism that arose directly from PNP might be associated with this patient’s corneal melting. However, the exact reason for the corneal melting and the relationship between CD-associated PNP and corneal melting remain to be elucidated.

CD is a rare neoplasm of lymphatic origin. The pathogenesis of CD-associated PNP remains unknown, although several hypotheses have been postulated, such as the secretion of autoantibodies from the tumor and the cross-reactivity of tumor antigens with epidermal antigens [2, 11]. Although a complete resection of a localized Castleman’s tumor could treat the disease and improve mucocutaneous lesions in most of patients [2, 7, 8], serum autoantibodies in some reports [12] and in this patient indeed remained detectable for as long as 2–4 years after surgery without recurrence of the tumor, suggesting that the immune reaction persisted. This might be the cause of the recurrence of mucosal lesions and the occurrence of corneal melting in this patient. It is necessary, therefore, to maintain effective immunosuppression for a prolonged postoperative period.

Currently, there is no standard therapy for conjunctival and corneal lesions from CD-associated PNP because of the rarity of this complication. As chronic cicatrizing conjunctivitis is more likely to occur in PNP, Tam et al. [13] proposed management with adequate lubrication, prophylactic antibiotics and corneal protection to reverse the underlying cause, to prevent symblepharon formation and to maintain the ocular surface. The successful local tacrolimus and cyclosporine treatment of corneal melting in the patient suggested that local immunosuppression in addition to systemic immunomodulatory therapy is critical for the treatment of corneal melting.

## Conclusion

This case highlights the potential for CD-associated PNP to present with corneal ulceration, which may lead to blindness along period after the complete excision of localized neoplasms. Systemic and local immunosuppression administrations are essential for the treatment of corneal complications. Further studies are needed to elucidate the exact mechanism of corneal melting in CD-associated PNP.

## Abbreviations

CD, Castleman’s disease; IOP, intraocular pressure; PNP, paraneoplastic pemphigus
